# Low Xanthophylls, Retinol, Lycopene, and Tocopherols in Grey and White Matter of Brains with Alzheimer’s Disease

**DOI:** 10.3233/JAD-220460

**Published:** 2023-06-27

**Authors:** C. Kathleen Dorey, Dennis Gierhart, Karlotta A. Fitch, Ian Crandell, Neal E. Craft

**Affiliations:** a Virginia Tech Carilion School of Medicine, Roanoke, VA, USA; b ZeaVision, Chesterfield, MO, USA; c Alzheimer’s Disease Research Center, Massachusetts General Hospital Boston, MA, USA; d Center for Biostatistics and Health Data Science, Virginia Tech, Roanoke, VA, USA; e Craft Technologies, Inc., Wilson, NC, USA

**Keywords:** Alzheimer’s disease, antioxidants, brain, carotenoids, deficiency, lutein, lycopene, *meso*-zeaxanthin, oxidation, tocopherols, zeaxanthin

## Abstract

**Background:**

Oxidative stress contributes to pathogenesis and progression of Alzheimer’s disease (AD). Higher levels of the dietary antioxidants— carotenoids and tocopherols— are associated with better cognitive functions and lower risk for AD, and lower levels of multiple carotenoids are found in serum and plasma of patients with AD. Although brains donated by individuals with mild cognitive impairment had significantly lower levels of lutein and beta-carotene, previous investigators found no significant difference in carotenoid levels of brains with AD and cognitively normal brains.

**Objective:**

This study tested the hypothesis that micronutrients are significantly lower in donor brains with AD than in healthy elderly brains.

**Methods:**

Samples of donor brains with confirmed AD or verified health were dissected into grey and white matter, extracted with organic solvents and analyzed by HPLC.

**Results:**

AD brains had significantly lower levels of lutein, zeaxanthin, anhydrolutein, retinol, lycopene, and alpha-tocopherol, and significantly increased levels of XMiAD, an unidentified xanthophyll metabolite. No meso-zeaxanthin was detected. The overlapping protective roles of xanthophylls, carotenes, α- and *γ*-tocopherol are discussed.

**Conclusion:**

Brains with AD had substantially lower concentrations of some, but not all, xanthophylls, carotenes, and tocopherols, and several-fold higher concentrations of an unidentified xanthophyll metabolite increased in AD (XMiAD).

## INTRODUCTION

Alzheimer’s disease (AD) is a progressive neurodegenerative disease estimated to affect six million Americans [1] and thirty-three million people worldwide [2]; large numbers of those affected are not yet diagnosed [1, 3]. Pathologic hallmarks in the brain are extracellular amyloid-β deposits and intracellular tangles of tau filaments, inflammation, and atrophy [1].

The disease process is multifactorial; factors increasing oxidative stress and biomarkers of oxidative stress correlate with cognitive impairment [4]. Current models of pathogenesis focus on mitochondrial dysfunction [5–7], disrupted autophagy of aged or damaged organelles, particularly mitochondria [8, 9], protein misfolding following S-nitrosylation or oxidation [10], or production of inflammatory cytokines and reactive oxygen species by microglia and astrocytes around amyloid plaques or neurofibrillary tangles [11]. All models include oxidative damage in the pathogenesis and progression of AD [12–14] and end with neuroinflammation and neuronal death [14, 15].

Neurons are highly susceptible to oxidative damage because of their high metabolic rates, tissue oxygen levels, stores of redox metals, and high membrane content of easily oxidized polyunsaturated fatty acids such as docosahexaenoic acid and arachidonic acid which together represent 20% of brain lipids. Because the brain operates in a delicate balance between its modest antioxidant reserve and the constant generation of damaging reactive species during normal functions, brain survival requires multiple forms of antioxidants [16]. Disruptions in this balance by increased oxidation or deficiency in brain antioxidants increase frailty [17], threaten functions essential to cognition, and may contribute to AD pathogenesis [13, 18].

Dietary xanthophylls and other carotenoids suppress key aspects of AD pathogenesis, including oxidation and inflammation [19–21], secretase activity and release of Aβ oligomers [22], and aggregation of Aβ fibrils [23]. Carotenoids have potential to modulate risk for AD or dementia, but low levels in AD brains have not been demonstrated. In fact, low plasma levels of xanthophylls in AD have been attributed to significantly lower concentrations of HDL in AD subjects who also have vascular complications [24]. Dietary tocopherols are important antioxidants that also inhibit oxidative stress and modulate proinflammatory pathways, apoptosis, and neuroprotective functions [25] that are implicated in risk for AD.

### Risk for dementia/AD

Those who closely followed the MIND diet (emphasizing higher intake of antioxidant-rich fruits, vegetables, legumes, nuts, and fish and minimal meat, dairy, and sweets) had significantly lower risk for AD [26, 27], better cognitive function prior to death, and less AD-related brain pathology [3]. Higher intake of total carotenoids or lutein/zeaxanthin over more than a decade was associated with almost 50% lower risk for an AD diagnosis and less global brain pathology; lutein/zeaxanthin intake was inversely correlated with AD diagnostic score, neuritic plaque severity, as well as neurofibrillary tangle density and severity [28]. Higher circulating levels of lutein and/or zeaxanthin and/or lycopene were associated with lower risk for dementia among 1,094 older French subjects [29] and lower risk for AD mortality in the NHANES study [30]. Greater dietary intake of carotenoids was correlated with better cognitive performance, lower risk for AD, and less severity of brain pathology in the Rush Memory and Aging Project [3, 28]. These observations are consistent with reports that those with higher levels of total carotenoids in diet, serum or plasma had slower cognitive decline [31], fewer white matter lesions [32, 33], less brain atrophy [34], and lower risk for AD diagnosis or less brain pathology [28, 35]. Higher gamma-tocopherol levels in AD brains were associated with lower Aβ and lower severity of neurofibrillary tangles [36]; higher levels were also associated with higher levels of six presynaptic proteins [37].

Despite its obvious relevance, little is known about carotenoid levels in brains with dementia or AD. Healthy elderly human brains contain more than 16 carotenoids and 66–77% of them are xanthophylls, dominated by β-cryptoxanthin, lutein, anhydrolutein, and zeaxanthin. [38]. Lutein and β-carotene concentrations were significantly lower in brains of elderly donors with mild cognitive impairment (MCI) than in those with normal cognition [39]. In contrast, donor brains with nominally identified AD had only slightly (nonsignificant) lower concentrations of all carotenoids [40]. This investigation tested the hypothesis that concentrations of lutein and zeaxanthin, one or more other carotenoid, and one or more tocopherols are lower in brains with confirmed AD than in healthy elderly brains of comparable age.

## MATERIALS AND METHODS

### Donor brains

Coded samples of frozen human brain were obtained from the Massachusetts Alzheimer’s Disease Research Center at the Massachusetts General Hospital, where the presence or absence of neuropathology in each sample was confirmed by microscopic examination. All study subjects or next of kin gave informed consent for the brain donation to MGH, where the protocol was approved by MGH Institutional Review Board. Upon receipt at the brain bank, the brains were dissected, and 0.5 cm sections were placed on cards, frozen on dry ice, and maintained at –80°C. Brain samples obtained from the Alzheimer’s Disease Research Center included regions in which AD lesions were thought to occur earlier and from regions thought to remain less affected by AD. Samples were shipped on dry ice and maintained at –80°C until extraction and analysis at Craft Technologies. The study of these samples was done in accord with the Helsinki Declaration of 1975 and approved by the Institutional Review Board of Schepens Eye Research, the Miami Miller School of Medicine at Florida Atlantic University, and Carilion Clinic.

The average age of all brains was 74.93±10.2 years; five brains (33.3%) were female and ten (66.6%) were male. The average age of healthy brains was 78.4±1.9 years as compared to 73.2±10.7 years average for the AD brains (N.S.). The percent of male donors was higher for brains with confirmed AD (70%) than for the healthy brains (60%). Average postmortem times were 12.1.±4.5 h for AD brains and 14.5±6.4 h for the healthy elderly (HE) brains (N.S.). Samples were retrieved from brain slices that had been stored at –80°C for prolonged periods (AD = 14.5±4.5 years; HE = 13.3±0.6 years, NS). Age, gender, and Broadman region of these samples are summarized in Table 1.

**Table 1:** Age, gender and Broadman region of donor brains confirmed as affected by Alzheimer ’s disease (AD) or as healthy elderly brains (HE)

Diagnosis	Gender	Age	Brain Area
AD	F	67	31
	F	88	4
	F	88	31
	F	86	24
	F	67	2
	F	88	6
	F	86	15
	M	69	31
	M	72	31
	M	56	30
	M	79	30
	M	80	28
	M	76	17-18
	M	59	17
	M	76	4
	M	69	6
	M	56	1–6
	M	56	3
	M	79	1
	M	80	1–6
	M	59	45-46
HE	F	85	30
	F	90	16
	F	85	6
	F	90	1
	M	73	31
	M	67	31
	M	77	17
	M	73	11
	M	67	44
	M	77	8-9

The data from HE brains were previously reported [38].

### Sample processing and analysis

Each human brain sample was dissected into white and grey matter, and a 1–3 g sample was weighed and placed in a mortar. The tissue was ground with a pestle immediately after adding approximately 0.5 g of sodium sulfate, and again after addition of 7 ml of hexane:ethyl acetate (90:10). The solvent was transferred to a glass funnel containing a glass fiber filter, and the filtrate collected in a 25 ml volumetric flask. The extraction was repeated until the volumetric was full. The extracts were dried under a stream of nitrogen gas. The dried extract was sonicated for 30 min in a mixture of 1 ml of 40% potassium hydroxide in methanol and 500μL of 10% pyrogallol in ethanol solution. Following addition of 2.5 ml of water and 5 ml of hexane:ethyl acetate solution, the mixture was vortexed for 45 s and the organic layer collected; the extraction was repeated two more times. The extract was washed twice with water and dried under nitrogen gas. The analytes were dissolved in 25μl of ethyl acetate and vortexed for 20 s, diluted with 75μl of mobile phase, vortexed again for 15 s, and sonicated for 15 s. The extract was transferred to a conical vial and centrifuged before HPLC, performed as previously described in detail [38, 41].

Xanthophyll composition was further examined in unsaponified brains extracted with combinations of organic solvents (tert-butyl methyl ether (MTBE), sodium dodecylsulfate, hexane, tetrohydrofuran), dried, re-dissolved in hexane/MTBE, and separated by normal HPLC using an ES Industries Diol 4×150 mm column and a hexane/dioxane gradient with tocol and β−apo-8’-carotenoate as standards. Carotenoids were measured by absorbance at 450 nm.

To determine meso-zeaxanthin content, brain samples extracted with MTBE were separated by normal phase chromatography using a Diol column with a hexane/dioxane gradient. The zeaxanthin fraction was collected, dried, diluted with the mobile phase (6% isopropanol in hexane), and separated by chiral HPLC using a Chiralpak AD 4.6×250 mm, 10 mm column and an isopropanol/hexane flow rate of 1.0 ml/min.

External standards used for quantification included: zeaxanthin, β-cryptoxanthin, and α-carotene (gifts from Hoffman-LaRoche (now DSM, Heerlen Netherlands); lutein (gift from Kemin Industries (Des Moines IA); lycopene and β-carotene from Sigma Chemical Co (St. Louis); retinol and α-tocopherol from US Biochemical (Cleveland OH), and tocol, *δ*- and *γ*-tocopherols (gift from Cognis Corporation, Chicago, IL). Response factors of lutein were used to quantify unidentified xanthophylls and 2’, 3’anhydrolutein; α-cryptoxanthin was quantified using the average of lutein and α-carotene response factors. Cis-isomers of lycopene and β-carotene were quantified using the response factors of their trans-isomers. Craft Technologies participated regularly in the National Institute of Standards and Technology (NIST) Micronutrient Quality Assurance Program for Fat-Soluble Vitamins.

The carotenoid content of HE brains was previously published [38].

### Statistical analysis

Concentrations of analytes in grey and white matter of AD and HE brains are presented as mean±S.E, expressed in pmol/g. Contributions of disease status, region, and brain matter to variance in analyte levels were analyzed by Analysis of Variance and Bonnferroni/Dunn posthoc tests (StatView; SAS Institute, Inc; Cary, NC). The ANOVA was performed on the full data set with samples from at least two regions for each brain, and every data point was associated with age, gender, AD or HE brain, grey or white matter, more or less vulnerable region. Region was consistently insignificant for all variables. Further comparisons of analyte levels in grey matter of HE and AD brains, and levels of lutein or zeaxanthin within a specific brain region were done by *t*-tests on the data set where regions were averaged so each brain had only two data points— one in grey matter and one in white matter. The initial analysis revealed that only lutein and zeaxanthin had significantly different levels in grey and white matter. For these reasons, the variables were compacted for further Analysis of Variance with repeated measures, so that measures of grey and white and different brain regions were treated as repeated measures from a donor brain. Because of the small number of women in the sample, gender was ignored.

To compare analyte deficiencies in AD brains, the mean concentration of each analyte concentration in HE grey matter and in HE white matter was determined. Then all analytes in grey matter of AD and HE brains were expressed as a percent of their HE means (% HE) in grey matter and repeated for white matter; the mean % HE was determined for each analyte in AD grey or white matter. This analysis was performed on a data set with averaged brain region so that each variable in a donor brain was represented by one data point in grey matter and one in white matter for each analyte.

Extremely high values for almost all analytes in the grey matter from two AD brains were excluded; these were statistically significant outliers for either HE or AD brain samples. Average values in the white matter of the same brain samples further indicated that these data were invalid; dehydration during specimen handling may have caused an artefactual increase in analyte concentrations in the grey matter.

Generalized linear mixed models with a logit link function and random intercepts per subject were used to model disease state as a function of the values of the analytes, separately in white and grey matter. Forward and backward stepwise selection was used to determine an appropriate set of predictors for each of these models. Support vector machines (SVMs, machine learning algorithms that analyze data for classification and regression analysis) were also used to predict disease state. The performance of the SVMs was evaluated using permutation testing with one-hundred random permutations examined.

Permutation testing involves randomly shuffling the labels for “HE” and “AD,” and evaluating the performance of the SVM on the randomly shuffled data. By randomizing the disease labels, we guarantee that any relationships observed between disease state and analytes in the permuted data are due solely to chance. Evaluating the SVM models on these permuted data sets allows us to estimate their performance under the null hypothesis, and by comparing that to their performance on the unpermuted data we can compute a *p*-value for the hypothesis of no relationship between disease state and the analyte values. Specifically, this *p*-value is the proportion of times the models fit the permuted data perform better than on the unpermuted data.

## RESULTS

**Fig. 1:**
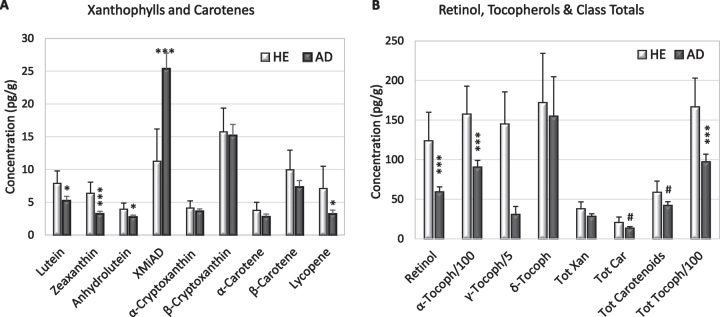
Mean concentrations (+S.E.) of xanthophylls and carotenes (A), retinol, and tocopherols (B) in HE and AD brains from (light and dark bars, respectively). B) Note different axis and that α-tocopherol and *γ*-tocopherol are represented as 1/100 and 1/5 their actual concentration. Significant differences between HE and AD determined by ANOVA: ^#^*p* = 0.07–0.09; ^*^p = 0.02–0.05; ^***^p = 0.002–0.004.

In both AD and HE brains, the most abundant xanthophyll and carotene were β-cryptoxanthin and β-carotene; both were exceeded in all brains by concentrations of retinol and α-, β-, and *γ*-tocopherols (Fig. 1). The concentrations of α-carotene or lycopene fell below the limits of detection in 28% of the specimens, but only 8% of brain specimens had undetectable levels of both carotenoids (Table 2).

**Table 2:** Xanthophylls in AD and HE donor brains and healthy elderly brains

Disease Status	ZEA	LUT	ANLUT	XMiAD	α-CRYPT	β-CRYPT	TOT. XAN
AD
Grey	4.2±0.5	7.3±1.0	2.9±0.4	26.9±3.9	3.7±0.5	18.1±3.1	65.0±7.7
White	2.4±0.4	3.5±0.6	2.6±0.4	20.4±3.2	3.5±0.5	12.7±1.5	46.7±5.5
G versus W *p*=	**0.009**	**0.0006**	*ns*	*ns*	*ns*	*ns*	*ns*
AD versus HE *p*=	**0.002**	**0.04**	**0.05**	**0.006**	*ns*	*ns*	*ns*
HE
Grey	8.0±1.6	10.0±1.7	4.0±0.6	7.0±2.4	3.8±0.6	16.0±2.2	52.8±6.6
White	4.8±1.7	5.7±1.9	4.0±1.2	*15.5*±6.4	4.5±1.4	15.4±4.8	54.2±17.3

Xanthophyll concentrations (Mean +SE in pmol/gram) in grey and white matter of donor HE and AD brains. *p*-values result from ANOVA with Bonferroni-Dunn posthoc tests. ZEA, zeaxanthin; LUT, lutein; ANLUT, anhydrolutein; XMiAD, xanthophyll metabolite increased in AD; α-CRYPT, alpha-Cryptoxanthin; β-CRYPT, β-cryptoxanthin; TOT. XAN, total xanthophylls.

### Comparisons of AD and HE brain micronutrients

AD brains had significantly lower concentrations of lutein (*p* = 0.03), zeaxanthin (*p* = 0.001), anhydrolutein (*p* = 0.05), lycopene (*p* = 0.05), retinol (*p* = 0.006), and α-tocopherol (*p* = 0.007); *γ*-tocopherol was significantly lower only in white matter (Fig. 1, Tables 2 and 3). Compared to HE brains, AD brains had higher levels of an unidentified peak with elution and spectral characteristics of a xanthophyll, referred to as xanthophyll metabolite increased in AD (XMiAD; *p* = 0.006; Table 2). Analysis of variance detected no difference in analyte content of brain regions thought to be affected earlier or later in AD progression.

**Table 3:** Carotenes, retinol, and tocopherol in AD and HE brains

Disease Status	α-CAR	β-CAR	LYC	TOT CARS	RETINOL	α-TOC /10	*γ*-TOC	*δ*-TOC	TOT TOC /10	TOT. CAROT
AD
Grey	3.2±0.6	6.7±1.2	3.6±0.9	13.5±2.5	62.6±7.6	901±115	712±250	242±104	997±143	78.4±9.8
White	2.4±0.5	8.0±.1.5	2.9±0.7	13.3±2.4	56.6±10.2	913±122	326±67	81±19	954±130	59.0±7.1
G versus W *p*=	*ns*	*ns*	*ns*	*ns*	*ns*	*ns*	*ns*	*ns*	ns	*ns*
AD versus HE *p*=	*ns*	*ns*	**0.05**	**T**	**0.003**	**0.003**	*ns*	*ns* **‡**	**0.004**	*ns*
HE
Grey	3.4±0.8	7.4±1.9	1.0±0.2	17.8±3.6	133±41	1435±212	375±157	155±49	1518±223	70.7±7.8
White	4.2±1.5	12.5±3.7	0.9±0.4	23.9±8.8	115±32	1716±435	775±248	189±76	1812±477	78.1±25.

Concentrations of analytes (Means±S.E., pmol/gram) in grey and white matter of donor brains. *p*-values result from ANOVA with Bonferroni-Dunn posthoc tests. α-CAR, α-carotene; β-CAR, β-carotene; LYC, lycopene; TOT CARS, total carotenes; α-TOC, α-tocopherol; *γ*-TOC, *γ*-tocopherol; *δ*-TOC, *δ*-tocopherol; TOT TOC, total tocopherols; TOT CAROT, total carotenoids. T: *p* = 0.07; ^‡^AD-HE difference was significant in white matter (*p* < 0.03).

### Lutein and zeaxanthin

**Fig. 2:**
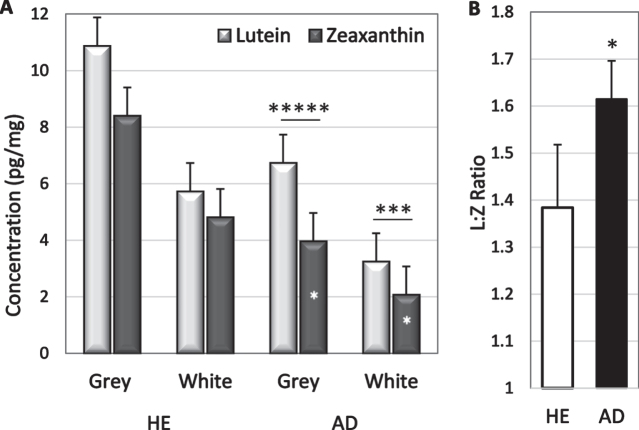
A) Lutein and zeaxanthin concentrations in grey and white matter of HE and AD brains. In AD brains, lutein exceeded zeaxanthin (* above bars); zeaxanthin in grey matter was lower in AD than in HE brains (*in bars). B) Mean L:Z ratio in AD and HE brains. ^*^p = 0.02–0.05; ^***^p = 0.002; ^****^p = 0.00002

Analyte concentrations were higher in grey matter than in white matter but only lutein and zeaxanthin had significantly higher concentrations in grey matter (*p* = 0.0006 and *p* = 0.009 respectively; Table 2). The greater abundance in grey matter was not significantly changed in disease; lutein concentration in grey matter of HE and AD brains was 1.8 and 2.0-fold higher than in white matter, and zeaxanthin was 1.7-and 1.8-fold higher (Table 2). Lutein concentrations exceeded zeaxanthin concentrations in both grey and white matter of AD brains (*p* = 0.003 and *p* = 0.03 respectively, Fig. 2a); sample size was inadequate to determine significance in HE brains. Zeaxanthin in both white and grey matter was lower in AD brains than in HE brains (*p* = 0.05 and 0.02, respectively). Lutein tended to be lower in white matter of AD brains (*p* = 0.08), but not in grey matter (Fig. 2). Healthy brains had 1.5-fold more lutein and 2-fold more zeaxanthin than AD brains suggesting a selective zeaxanthin deficit in AD brains. In fact, the lutein to zeaxanthin ratio was higher in AD brains than in HE brains (Fig. 2b).

### Absence of meso-zeaxanthin

**Fig. 3:**
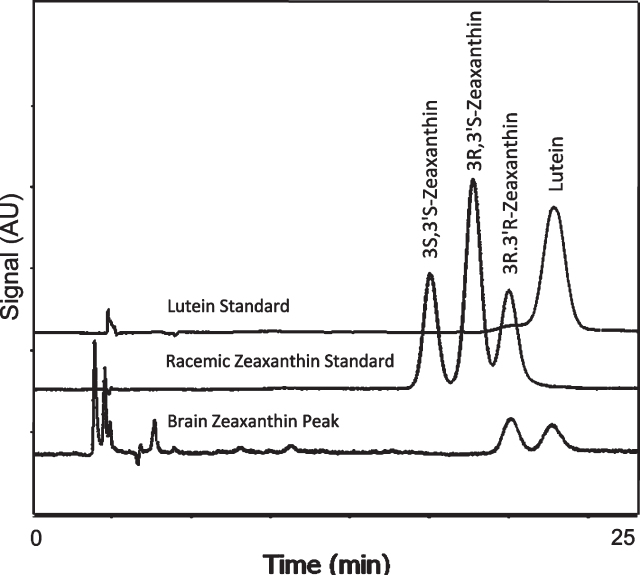
Separation of zeaxanthin optical isomers in a racemic mixture and in the brain zeaxanthin fraction collected during normal phase LC. Brain contained only the 3R, 3’R enantiomer of zeaxanthin.

Chiral analysis of the zeaxanthin fraction isolated from unsaponified human brain revealed only the 3R-3R’-zeaxanthin found in the normal diet and contaminating lutein. Meso-zeaxanthin (3R-3’S-zeaxanthin) was not detected (Fig. 3).

### Analyte deficiencies in AD brains

To compare relative deficiency of analytes, the mean concentration of each analyte in HE grey and white matter was determined. Then all analytes in grey matter of AD and HE brains were expressed as a percent of their mean in HE grey matter, and repeated for white matter, and the mean % HE in AD grey matter and white matter was determined for each analyte.

**Fig. 4:**
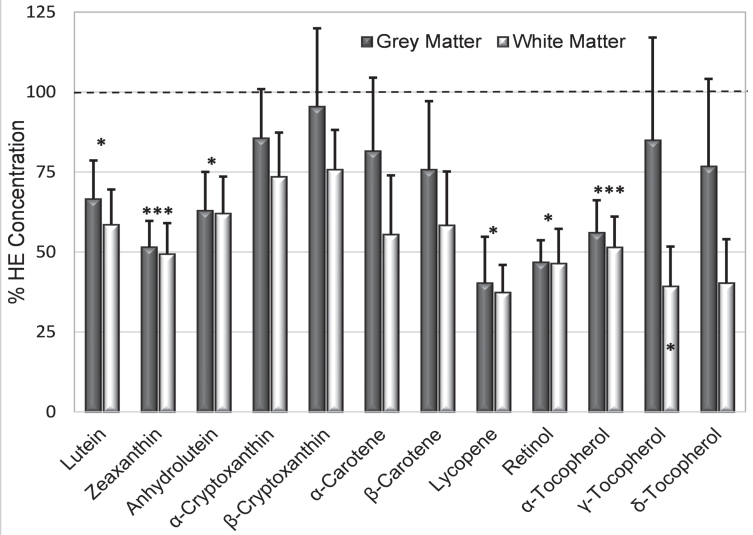
Mean % HE (+S.E.) illustrates relative analyte deficits in AD grey and white matter (dark and light bars, respectively). Asterisks above the bar identify analytes whose % HE in AD brains was significantly below those in HE brains (dashed line); *γ*-tocopherol was significantly lower only in grey matter (asterisk in the bar). ^*^p = 0.02–0.05; ^***^p = 0.001–0.004

The greatest deficit in AD brains was lycopene (mean only 38.8 % HE, *p* = 0.04), followed in order by retinol (46.5 % HE, *p* = 0.003), zeaxanthin (48.9 % HE, *p* = 0.002), alpha-tocopherol (53.7 % HE, *p* = 0.003), anhydrolutein and lutein (both 62 % HE); *γ*-tocopherol was significantly reduced only in white matter (Fig. 4). Smaller deficits in other analytes were not significant. Deficiencies in zeaxanthin and retinol in AD grey matter were striking as only 6% of samples had concentrations that reached the means in HE brains. In contrast, concentration of lycopene (the most deficient analyte in AD brains) was very low or undetectable in half of AD grey matter samples while the concentration in the upper third of samples was higher than the mean lycopene concentration in HE grey or white matter. Almost all analyte concentrations were more depressed in white matter than in grey matter (Fig. 4). For all but five analytes (α- and β-cryptoxanthin, α-carotene, and *γ*- and *δ*-tocopherol), the mean concentration in AD grey matter was more than 25% below the mean concentrations in HE grey matter (Fig. 4). Analyte concentrations in AD grey matter were variable; 38–50% of AD brains had individual analytes with a concentration that was greater or equal to their average in HE brains.

### Brain xanthophyll metabolite increased in AD

**Fig. 5:**
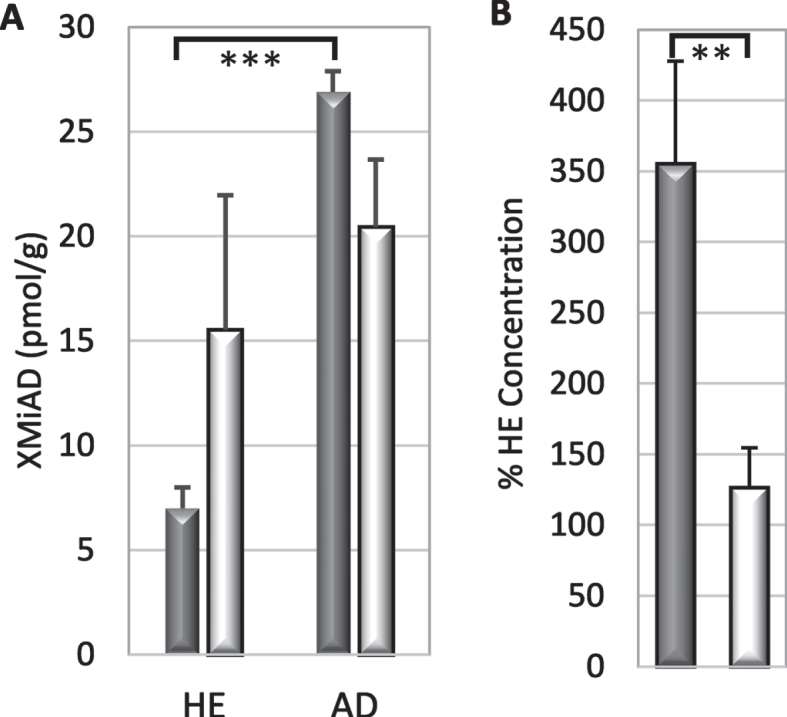
A) XMiAD concentration (mean + S.E.) in grey and white matter (dark and light bars, respectively) of HE and AD brains (*p* = 0.002; ANOVA). B) Relative to HE brains, the fold-increase of XMiAD in AD was greater in grey matter than in white matter (*p* = 0.007).

XMiAD was the last of eight small unidentified peaks with elution and absorption characteristics of xanthophylls; the first seven small peaks had concentrations that were 10–50% lower in AD brains (data not shown). XMiAD was intriguing because it was the only analyte whose concentration was higher in AD brains. XMiAD was found in both HE and AD brains but its concentration (estimated from the lutein absorption coefficient) was significantly higher in AD brains (*p* = 0.006, ANOVA, Table 2). B XMiAD concentration in AD grey matter was significantly higher than that in HE grey matter (*p* = 0.002, two-tailed T-test) but the difference in white matter was not significant (Fig. 5A). Moreover, AD grey matter had 3.5-fold more XMiAD than HE grey matter, which was significantly larger than the 1.3-fold higher XMiAD in white matter of AD brains (*p* = 0.007; Fig. 5B).

**Fig. 6:**
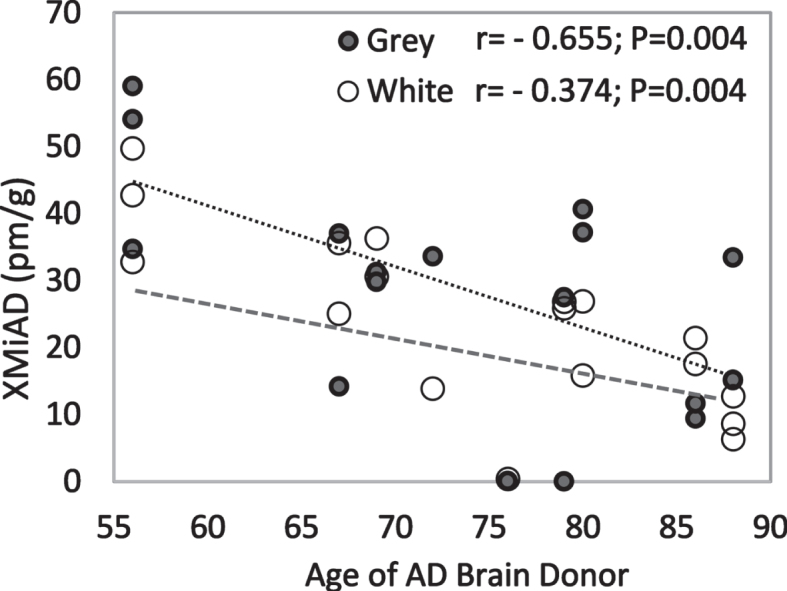
Negative correlation of XMiAD with age of AD brain donors in both grey and white matter (dark and light markers, respectively).

XMiAD concentration declined significantly with increasing age in all AD brains (*r* = –48; *p* = 0.002) and in grey matter and white matter analyzed separately (Fig. 6). In grey and white matter of both HE and AD brains, XMiAD was significantly correlated with *γ*-tocopherol as well as retinol and *δ*-tocopherol, although less strongly (Table 4). XMiAD was correlated with zeaxanthin, anhydrolutein, and β-cryptoxanthin only in white matter of AD brains (Table 4).

**Table 4:** XMiAD correlations with age and other analytes

	Healthy Elderly		Alzheimer’s Disease	
	Brains (*r* values)		Brains (*r* values)	
Analyte	Grey	White	Grey	White
Lutein	0.05	0.53	0.41	0.37
Zeaxanthin	0.19	0.40	0.38	0.50^a^
Anhydrolutein	0.17	0.44	0.54^a^	0.61^b^
β-Cryptoxanthin	–0.23	0.51	0.20	0.63^b^
Lycopene	0.17	0.59	0.33^a^	0.40
β-Carotene	–0.59	0.64^a^	0.21	0.37
Retinol	0.72^a^	0.79^b^	0.48^a^	0.70^c^
α-Tocopherol	0.69	0.30	0.56	0.49
*γ*-Tocopherol	0.90^c^	0.95^d^	0.51^‡a^	0.68^‡c^
*δ*-Tocopherol	0.88^c^	0.76^a^	0.20	0.53^a^
Total Xanthophylls	0.28	0.77^b^	0.40	0.48^b^

Superscripts represent p values: a = 0.05-0.01; b = 0.009-0.001; c = 0.00001-0.0005; d = 0.00000005; ^‡^Correlation with log *γ*-tocopherol.

### Models to predict disease status

Attempts were made to identify analytes most strongly related to the presence of AD in the brain, though none yielded meaningful results. Due to the large number of analytes compared to the sample size, the generalized linear mixed models were able to identify a combination of the analyte values which perfectly distinguished AD and HE brains. Because this phenomenon violates the assumptions for the linear models, the approach was found to be invalid. Support vector machines designed to find such a linear combination [42] were able to predict disease state with 100% accuracy. However, permutation testing revealed that accurate prediction was still achieved when the labels for disease state were randomly shuffled (*p* = 0.8). In both cases, the null results are likely due to lack of power because of the small sample size in relation to the number of analytes explored.

## DISCUSSION

The main findings of this hypothesis-generating study are that the concentrations of lutein, zeaxanthin, lycopene, retinol, and α-tocopherol are profoundly lower in brains with documented AD, that lycopene and zeaxanthin were the two most deficient antioxidants, and that AD brains had significantly higher levels of XMiAD, a yet unidentified xanthophyll. To our knowledge, this is the first report comparing levels of retinol, carotenoids, and tocopherols in brains with confirmed AD neuropathology with those in healthy elderly brains. These data do not permit us to assess why these analyte levels are low, but the data are consistent with existing lines of evidence [4, 28, 37, 39, 43, 44].

Inadequate levels of brain carotenoids would reduce the potential neuroprotection [45] offered by their antioxidant [46], anti-inflammatory [46–49], and anti-amyloidogenic activities [23]. Reduced trapping of peroxynitrite by carotenoids [24, 50] would increase risk of lipid peroxidation, protein nitration, and mitochondrial dysfunction that contribute to neuronal death [51].

Lutein/zeaxanthin and lycopene deficiencies in these AD brains are consistent with previous findings. Brain neuropathology typical of AD and risk of AD mortality were inversely related to dietary levels of lutein/zeaxanthin and lycopene in the Memory and Aging Project [28], and serum lutein, zeaxanthin, and lycopene in the NHANESIII study [30]. Poor cognitive performance among 589 elderly adults in the EVA study was linked to low plasma zeaxanthin and lycopene [52], the two most deficient carotenoids in AD brains.

Donor brains from centenarians with MCI had significantly lower levels of lutein and β-carotene, but not lycopene or zeaxanthin, which were low but not significant [39]. Interestingly, lycopene was the most deficient analyte in the MCI brains with a concentration that was only 44.3% of the mean in cognitively normal brains— equivalent to the lycopene deficiency in AD brains (40.3 and 37.4% HE in grey and white matter, respectively). Greater variation in lycopene levels in MCI brains than in AD brains could explain why statistical significance was not achieved; the standard error represented 36% of the mean lycopene concentration in the MCI brains, whereas it was 25% and 24.1% in AD brains. Similarly, the SEM for β-carotene in these HE brains was 36.7% and 30.0% of mean β-carotene in grey and white matter, thus contributing to lack of a significant difference in β-carotene content in AD brains.

The importance of low lutein and zeaxanthin in AD brains is emphasized by reports that those with highest levels of lutein and zeaxanthin in their diet, plasma, serum, or macular pigment (retinal accumulation of lutein and zeaxanthin) have a 50% lower risk for an AD diagnosis [28, 53], and higher cognitive performance [43, 54–58]. Performance of cognitively normal and AD subjects on the Mini-Mental State Examination correlated positively with plasma levels of both lutein and zeaxanthin, even after correction for HDL cholesterol levels [24]. The observation that lutein and zeaxanthin were selectively lower in white matter aligns with previous reports associating better white matter integrity with higher serum concentrations of lutein and zeaxanthin [35], and increased risk for white matter lesions with low serum carotenoids [32]. Presence of white matter lesions are a risk factor for greater progression from MCI to AD [59, 60] and white matter dysfunction is linked to cognitive decline in mutation carriers [61]. Lutein and zeaxanthin may slow cognitive decline and reduce risk for AD [28, 62] by helping to preserve white matter connectivity.

### Meso-zeaxanthin not detected

The absence of meso-zeaxanthin in the brain is not surprising, as it is rarely detected in human serum except in subjects taking meso-zeaxanthin supplements [63, 64] or eating eggs laid by meso-zeaxanthin supplemented chickens [65]. Other known sources— fish skin, shrimp shells, turtle fat, and vertebrate eyes [66]— are rare dietary components. A small trial found that absorption of lutein, zeaxanthin, and meso-zeaxanthin from a combined supplement was less robust than when the supplement contained equal amounts of lutein and zeaxanthin alone or in combination [63]; this raises the possibilities that meso-zeaxanthin in the diet would be poorly absorbed, and/or that meso-zeaxanthin could interfere with absorption of lutein and zeaxanthin.

Meso-zeaxanthin found in macular pigment is produced by conversion of lutein to meso-zeaxanthin by the enzyme RPE65 in the retinal pigment epithelium (RPE, a monolayer located under the retina) [66]. RPE65 is specifically localized in the RPE where it also plays an essential role in the visual cycle [66]. Zeaxanthin and meso-zeaxanthin are accumulated in macula pigment by binding to GSTP1 (glutathione-S-transferase, pi isoform) where they absorb damaging blue light and synergistically reduce membrane peroxidation [67–69]; the bound meso-zeaxanthin is thus not available for circulation to the brain.

### Brain tocopherols and AD risk

The almost 50% lower level of α-tocopherol in grey and white matter and more than 50% lower *γ*-tocopherol in white matter would have significant effects on brain health, but it is worth noting that even HE brains may have inadequate α- and *γ*-tocopherol; 60% of Irish community-dwelling seniors had inadequate vitamin E in their diet [70]. Higher dietary intake of α- and *γ*-tocopherols were independently associated with lower risk for AD in the Chicago Health and Aging Project [71]. Subsequent studies found that higher brain *γ*-tocopherol (but not α-tocopherol) was associated with lower amyloid load and lower neurofibrillary tangle severity in AD brains [36], higher levels of presynaptic protein and lower levels of activated microglia in the cortex [37, 72]. Risk for AD is associated with dietary tocopherol, predominantly *γ*-tocopherol [73]; unfortunately, supplementation with α-tocopherol reduces *γ*-tocopherol in serum [74] and probably brain [36]. *γ*-Tocopherol— but not α-tocopherol— traps highly reactive oxidant peroxynitrite [75] forming a nitration product (5-nitro-*γ*-tocopherol) that accumulates in AD brains [76].

### Brain carotenoids correlate with cognition

Low brain carotenoid levels would also contribute to functional decline in cognition, which has been linked to lutein and zeaxanthin in diet, plasma, or macular pigment in children [77], older adults [57], and octogenarians and centenarians [39]. Prenatal levels of lutein/zeaxanthin in maternal diets were associated with better verbal intelligence in their offspring [78]. Large population studies with thousands of subjects in the US, France, and Ireland have confirmed higher levels of lutein and zeaxanthin in their diet [43], plasma [52, 56], or macular pigment [58] were associated with higher scores on cognitive tests and higher processing speed.

Carotenoid supplement studies confirm a direct effect of carotenoids on brain function. Improved cognitive performance was observed after 3–18 months supplementation with β-carotene [79], lutein and docosahexaenoic acid [80], lutein and zeaxanthin [81, 82], as well as lutein, meso-zeaxanthin, and zeaxanthin [83, 84]. Supplementation with lutein and zeaxanthin increased visual processing speeds in young adults [85] and altered brain activation patterns in older adults [86, 87].

### Carotenoids modulate risk for AD

Risk for diagnosis of AD was higher in those with low dietary intake of total carotenoids or lutein/zeaxanthin, specifically [28]. Moreover, subjects with AD had low plasma or serum levels of carotenoids and tocopherols [44, 88]. Premortem serum levels of carotenoids and tocopherols were significantly correlated with those in temporal but not occipital lobes of donor brains [89]. In the Three-City Bordeaux study, repeated brain imaging of 461 non-demented participants over ten years revealed smaller loss of temporal lobe volume in those with higher plasma total carotenoids [34]. Thus, it is not surprising that each of the carotenoids found low in these AD brains was reported previously to be low in serum or patients with AD (Table 5). In general, analytes that were significantly lower in these AD brains were also low in the MCI brains (Table 5, compare studies 1 and 3). β-carotene was significantly lower in the MCI brains but not in AD brains, and the reverse was true for lycopene. Low lycopene was reported in these AD brains and five studies of AD plasma or sera (Table 5, studies 1, 4,5,7,8,9); lower β-carotene was significant in MCI brains and five AD studies (Table 5, studies 4, 5, 6, 8, 12, 13). Curiously, twelve studies reported low lycopene and/or low β-carotene in brains with MCI or serum or plasma of patients with AD, but only two found significantly low levels of both (Table 5, Study 5 and 8). The possible effect of different diets cannot be discounted, but differences in which specific analytes were significantly lower in these small brain studies may disappear (or be affirmed) in larger studies.

**Table 5:** Comparison of micronutrients associated with AD or MCI in this report and the literature

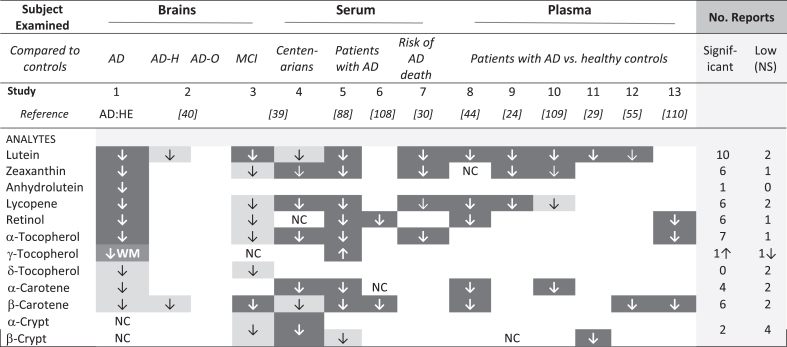

AD:HE, these data; AD-H, hippocampus; AD-O, Occipital lobe; NC, no change; WM, white matter. Dark cells identify significantly lower analyte concentration, lighter boxes identify analytes that were reported low, but not significant. Open cells indicate an analyte was not detected or not mentioned.

A previous investigation comparing micronutrients in brains with AD and those with normal cognition did not detect a significant difference in carotenoid or tocopherol content of the occipital or hippocampal regions, although AD hippocampal lutein and β-carotene concentration were lower [40]. The absence of significant differences may relate to the small sample size, or the true status of the brains obtained from NDRI, where information about brain health or presence of AD was obtained from the donor or family; brains considered cognitively normal may have had AD pathology and cognitive impairment, and brains considered to have AD may include other forms of dementia. For example, among over 100,000 French patients with early dementia, 40% were attributed to alcohol-related brain damage [90].

A xanthophyll and a carotene were the two most deficient carotenoids in both AD brains (zeaxanthin and lycopene) and MCI brains (lutein and β-carotene). Xanthophyll-carotene pairs act synergistically to prevent lipid oxidation [91]. Moreover, xanthophylls and carotenes modulate different protective signaling pathways. Carotenes and retinoids signal through nuclear receptors RAR/RXR or RAR/PPAR to regulate inflammation, the immune system, as well as axonal and neurite regeneration [92, 93]. Lutein and zeaxanthin activate nuclear-factor erythroid 2-related factor 2 (Nrf-2) to upregulate phase II protective enzymes such as heme oxygenase-1, superoxide dismutase, peroxiredoxin, and glutathione-S-transferase, pi isoform (GSTPI) [94, 95]. Peroxiredoxin 6 and GSTPI form a complex that repairs peroxidized phospholipids in cell membranes [96, 97]. Zeaxanthin binds to GSTP1, resulting in synergistic protection against membrane lipid peroxidation [68]. Biomarkers of lipoperoxidation are well known in AD and MCI brains [98]; GSTPI variants increase risk for AD [99]. Thus, zeaxanthin may play a unique role in repairing oxidative damage to mitochondria that contributes to neuronal death by apoptosis or ferroptosis [51]. Together these studies suggest that xanthophyll, carotene, and tocopherol antioxidants may play both distinct and overlapping roles in maintaining brain health.

### XMIAD, a possible xanthophyll metabolite

XMiAD accumulation in AD brains was an unexpected observation. The absorption spectrum of XMiAD was clearly that of a xanthophyll and it eluted among xanthophylls, but its elution position does not match xanthophylls previously observed in human serum or tissues. Unfortunately, we lacked sufficient sample for further analysis. We propose that it is a xanthophyll metabolite derived from an oxidation event. Oxidized xanthophylls and carotenoids form adducts with a wide range of radicals, forming nitrocarotenoids such as nitrozeaxanthin and nitrolutein (derived from interaction with peroxynitrite) [50, 101–104], 3-methoxyzeaxanthin,identified in human macular pigment [105], as well as sulfonyl and thiyl adducts [106, 107]. The strong positive correlation of XMiAD with *γ*-tocopherol in both grey and white matter (*r* = 0.9 and 0.95; Table 4) suggests that levels of both may respond to levels of highly reactive peroxynitrite; a positive correlation could indicate that the reaction producing XMiAD protects gamma-tocopherol. This seems unlikely as carotenoids are less effective than tocopherols in inhibiting lipid peroxidation caused by free radicals [100]. It is more likely that XMiAD is an oxidation-derived adduct protected by *γ*-tocopherol. Further investigation of this intriguing metabolite is clearly needed.

Previous studies of analytes in healthy brains [39, 40, 89] reported higher analyte concentrations than we found in the healthy elderly brains. After comparing ages of the brains, extraction procedures, and analysis methods, we conclude that the likely explanation for the difference in analyte concentrations is that these AD and HE brains were stored at –80°C for years before the brain samples were analyzed. The observed difference between these AD and HE brains is not related to storage time as both sets were stored for extended times.

Strengths of this small study include confirmation of the presence of AD and absence of pathology by neuropathologists, that HE and AD brains were obtained and analyzed simultaneously, and that data were obtained from both grey and white matter. Limitations include the small sample size, the cross-sectional design, the inability to adjust for other factors known to influence AD (e.g., nutrition, exercise, cardiovascular health, social interactions), and the impossibility to discern whether low analytes contributed to AD, declined with oxidative processes associated with disease progression, or reflect lower dietary intake or absorption of nutrients.

### Implications of these data

Given the vulnerability of the brain to oxidative damage, constant generation of reactive species through normal brain functions, and limited antioxidant resources [4, 16], it is reasonable to consider that brains with 40–50% lower levels of α-tocopherol and four carotenoids, and marginally lower levels in three of the four remaining brain carotenoids would be more vulnerable to oxidative damage and inflammation [13]. This new evidence of selective carotenoid and tocopherol deficiencies in the brains of subjects with AD add further support to the growing evidence (recently reviewed by Polidori et al. [4]) that greater dietary intake of lutein, zeaxanthin, lycopene as well as α- and *γ*-tocopherols may slow cognitive decline prior to— and possibly following— a diagnosis with AD.

### Conclusion

In conclusion, we found that concentrations of lycopene, retinol, zeaxanthin, α-tocopherol, anhydrolutein, and lutein were all strikingly lower in brains with confirmed neuropathology characteristic of AD than in HE brains with no evidence of pathology. Meso-zeaxanthin was not detected. Only XMiAD, an unidentified xanthophyll, had significantly higher concentration in AD brains than in HE brains. Further studies are needed to confirm these results in another population and to identify XMiAD.

## References

[1] (2020) 2020 Alzheimer’s disease facts and figures. Alzheimers Dement 113, 200–208. 10.1002/alz.12068 32157811

[2] World Health Organization, Dementia, https://www.who.int/news-room/fact-sheets/detail/dementia, Accessed March 21, 2022.

[3] Dhana K , James BD , Agarwal P , Aggarwal NT , Cherian LJ , Leurgans SE , Barnes LL , Bennett DA , Schneider JA (2021) MIND diet, common brain pathologies, and cognition in community-dwelling older adults. J Alzheimers Dis 83, 683–692. 34334393 10.3233/JAD-210107 PMC8480203

[4] Polidori MC , Stahl W , Griffiths HR (2021) Nutritional cognitive neuroscience of aging: Focus on carotenoids and cognitive frailty. Redox Biol 44, 101996. 34090844 10.1016/j.redox.2021.101996 PMC8212151

[5] Gowda P , Reddy PH , Kumar S (2022) Deregulated mitochondrial microRNAs in Alzheimer’s disease: Focus on synapse and mitochondria. Ageing Res Rev 73, 101529. 34813976 10.1016/j.arr.2021.101529 PMC8692431

[6] Medala VK , Gollapelli B , Dewanjee S , Ogunmokun G , Kandimalla R , Vallamkondu J (2021) Mitochondrial dysfunction, mitophagy, and role of dynamin-related protein 1 in Alzheimer’s disease. J Neurosci Res 99, 1120–1135. 33465841 10.1002/jnr.24781

[7] Bhatia V , Sharma S (2021) Role of mitochondrial dysfunction, oxidative stress and autophagy in progression of Alzheimer’s disease. J Neurol Sci 421, 117253. 33476985 10.1016/j.jns.2020.117253

[8] Festa BP , Barbosa AD , Rob M , Rubinsztein DC (2021) The pleiotropic roles of autophagy in Alzheimer’s disease: From pathophysiology to therapy. Curr Opin Pharmacol 60, 149–157. 34419832 10.1016/j.coph.2021.07.011 PMC8519395

[9] Tran AP , Warren PM , Silver J (2020) Regulation of autophagy by inhibitory CSPG interactions with receptor PTPsigma and its impact on plasticity and regeneration after spinal cord injury. Exp Neurol 328, 113276. 32145250 10.1016/j.expneurol.2020.113276 PMC7145755

[10] Nakamura T , Oh CK , Zhang X , Lipton SA (2021) Protein S-nitrosylation and oxidation contribute to protein misfolding in neurodegeneration. Free Radic Biol Med 172, 562–577. 34224817 10.1016/j.freeradbiomed.2021.07.002 PMC8579830

[11] Serrano-Pozo A , Mielke ML , Gomez-Isla T , Betensky RA , Growdon JH , Frosch MP , Hyman BT (2011) Reactive glia not only associates with plaques but also parallels tangles in Alzheimer’s disease. Am J Pathol 179, 1373–1384. 21777559 10.1016/j.ajpath.2011.05.047 PMC3157187

[12] Nunomura A , Perry G , Aliev G , Hirai K , Takeda A , Balraj EK , Jones PK , Ghanbari H , Wataya T , Shimohama S , Chiba S , Atwood CS , Petersen RB , Smith MA (2001) Oxidative damage is the earliest event in Alzheimer disease. J Neuropathol Exp Neurol 60, 759–767. 11487050 10.1093/jnen/60.8.759

[13] Ionescu-Tucker A , Cotman CW (2021) Emerging roles of oxidative stress in brain aging and Alzheimer’s disease. Neurobiol Aging 107, 86–95. 34416493 10.1016/j.neurobiolaging.2021.07.014

[14] Buccellato FR , D’Anca M , Fenoglio C , Scarpini E , Galimberti D (2021) Role of oxidative damage in Alzheimer’s disease and neurodegeneration: From pathogenic mechanisms to biomarker discovery. Antioxidants (Basel) 10, 10091353. 10.3390/antiox10091353 PMC8471953 34572985

[15] Uddin MS , Kabir MT , Jalouli M , Rahman MA , Jeandet P , Behl T , Alexiou A , Albadrani GM , Abdel-Daim MM , Perveen A , Ashraf GM (2022) Neuroinflammatory signaling in the pathogenesis of Alzheimer’s disease. Curr Neuropharmacol 20, 126–146. 34525932 10.2174/1570159X19666210826130210 PMC9199559

[16] Cobley JN , Fiorello ML , Bailey DM (2018) 13 reasons why the brain is susceptible to oxidative stress. Redox Biol 15, 490–503. 29413961 10.1016/j.redox.2018.01.008 PMC5881419

[17] Polidori MC , Mecocci P (2022) Modeling the dynamics of energy imbalance: The free radical theory of aging and frailty revisited. Free Radic Biol Med 181, 235–240. 35151828 10.1016/j.freeradbiomed.2022.02.009

[18] Nunomura A , Tamaoki T , Motohashi N , Nakamura M , McKeel DW Jr , Tabaton M , Lee HG , Smith MA , Perry G , Zhu X (2012) The earliest stage of cognitive impairment in transition from normal aging to Alzheimer disease is marked by prominent RNA oxidation in vulnerable neurons. J Neuropathol Exp Neurol 71, 233–241. 22318126 10.1097/NEN.0b013e318248e614 PMC3288284

[19] Grodzicki W , Dziendzikowska K (2020) The role of selected bioactive compounds in the prevention of Alzheimer’s disease. Antioxidants (Basel) 9, 9030229. 10.3390/antiox9030229 PMC7139322 32168776

[20] Kaulmann A , Bohn T (2014) Carotenoids, inflammation, and oxidative stress–implications of cellular signaling pathways and relation to chronic disease prevention. Nutr Res 34, 907–929. 25134454 10.1016/j.nutres.2014.07.010

[21] Che H , Li Q , Zhang T , Wang D , Yang L , Xu J , Yanagita T , Xue C , Chang Y , Wang Y (2018) Effects of astaxanthin and docosahexaenoic-acid-acylated astaxanthin on Alzheimer’s disease in APP/PS1 double-transgenic mice. J Agric Food Chem 66, 4948–4957. 29695154 10.1021/acs.jafc.8b00988

[22] Sho M , Ichiyanagi N , Imaizumi K , Ishikawa M , Morimoto S , Watanabe H , Okano H (2020) A combinational treatment of carotenoids decreases Abeta secretion in human neurons via beta-secretase inhibition. Neurosci Res 158, 47–55. 31606373 10.1016/j.neures.2019.10.006

[23] Lakey-Beitia J , Kumar DJ , Hegde ML , Rao KS (2019) Carotenoids as novel therapeutic molecules against neurodegenerative disorders: Chemistry and molecular docking analysis. Int J Mol Sci 20, 20225553. 10.3390/ijms20225553 PMC6888440 31703296

[24] Dias IH , Polidori MC , Li L , Weber D , Stahl W , Nelles G , Grune T , Griffiths HR (2014) Plasma levels of HDL and carotenoids are lower in dementia patients with vascular comorbidities. J Alzheimers Dis 40, 399–408. 24448787 10.3233/JAD-131964 PMC4230763

[25] Ungurianu A , Zanfirescu A , Nitulescu G , Margina D (2021) Vitamin E beyond its antioxidant label. Antioxidants (Basel) 10, 10050634. 10.3390/antiox10050634 PMC8143145 33919211

[26] Morris MC , Tangney CC , Wang Y , Sacks FM , Barnes LL , Bennett DA , Aggarwal NT (2015) MIND diet slows cognitive decline with aging. Alzheimers Dement 11, 1015–1022. 26086182 10.1016/j.jalz.2015.04.011 PMC4581900

[27] Morris MC , Tangney CC , Wang Y , Sacks FM , Bennett DA , Aggarwal NT (2015) MIND diet associated with reduced incidence of Alzheimer’s disease. Alzheimers Dement 11, 1007–1014. 25681666 10.1016/j.jalz.2014.11.009 PMC4532650

[28] Yuan C , Chen H , Wang Y , Schneider JA , Willett WC , Morris MC (2020) Dietary carotenoids related to risk of incident Alzheimer dementia (AD) and brain AD neuropathology: A community-based cohort of older adults. Am J Clin Nutr 113, 200–208. 10.1093/ajcn/nqaa303 PMC7779228 33184623

[29] Feart C , Letenneur L , Helmer C , Samieri C , Schalch W , Etheve S , Delcourt C , Dartigues JF , Barberger-Gateau P (2016) Plasma carotenoids are inversely associated with dementia risk in an elderly French cohort. J Gerontol A Biol Sci Med Sci 71, 683–688. 26286605 10.1093/gerona/glv135

[30] Min JY , Min KB (2014) Serum lycopene, lutein and zeaxanthin, and the risk of Alzheimer’s disease mortality in older adults. Dement Geriatr Cogn Disord 37, 246–256. 24247062 10.1159/000356486

[31] Kesse-Guyot E , Andreeva VA , Ducros V , Jeandel C , Julia C , Hercberg S , Galan P (2014) Carotenoid-rich dietary patterns during midlife and subsequent cognitive function. Br J Nutr 111, 915–923. 24073964 10.1017/S0007114513003188

[32] Den Heijer T , Launer LJ , De Groot JC , De Leeuw FE , Oudkerk M , Van Gijn J , Hoffman A , Breteler MMB (2001) Serum carotenoids and cerebral white matter lesions: The Rotterdam scan study. J Am Geriatrics Soc 49, 642–646. 10.1046/j.1532-5415.2001.49126.x 11380759

[33] Ohshima Y , Mizuno T , Yamada K , Matsumoto S , Nagakane Y , Kondo M , Kuriyama N , Miyazaki T , Takeda K , Nishimura T , Nakagawa M , Ozasa K , Watanabe Y (2013) Low vitamin and carotenoid levels are related to cerebral white matter lesions. J Nutr Health Aging 17, 456–460.2363654710.1007/s12603-012-0419-z

[34] Thomas A , Proust-Lima C , Baillet M , Helmer C , Delcourt C , Foubert-Samier A , Catheline G , Feart C , Samieri C (2021) Plasma carotenoids and medial temporal lobe atrophy in older adults. Clin Nutr 40, 2460–2463. 33558180 10.1016/j.clnu.2020.09.056

[35] Mewborn CM , Terry DP , Renzi-Hammond LM , Hammond BR , Miller LS (2018) Relation of retinal and serum lutein and zeaxanthin to white matter integrity in older adults: A diffusion tensor imaging study. Arch Clin Neuropsychol 33, 861–874. 29161349 10.1093/acn/acx109

[36] Morris MC , Schneider JA , Li H , Tangney CC , Nag S , Bennett DA , Honer WG , Barnes LL (2015) Brain tocopherols related to Alzheimer’s disease neuropathology in humans. Alzheimers Dement 11, 32–39. 24589434 10.1016/j.jalz.2013.12.015 PMC4148466

[37] de Leeuw FA , Honer WG , Schneider JA , Morris MC (2020) Brain gamma-tocopherol Levels are associated with presynaptic protein levels in elderly human midfrontal cortex. J Alzheimers Dis 77, 619–627. 32741813 10.3233/JAD-200166 PMC7592653

[38] Craft NE , Haitema TB , Garnett KM , Fitch KA , Dorey CK (2004) Carotenoid, tocopherol, and retinol concentrations in elderly human brain. J Nutr Health Aging 8, 156–162. 15129301

[39] Johnson EJ , Vishwanathan R , Johnson MA , Hausman DB , Davey A , Scott TM , Green RC , Miller LS , Gearing M , Woodard J , Nelson PT , Chung HY , Schalch W , Wittwer J , Poon LW (2013) Relationship between serum and brain carotenoids, alpha-tocopherol, and retinol concentrations and cognitive performance in the oldest old from the Georgia centenarian study.. J Aging Res 2013, 951786. 23840953 10.1155/2013/951786 PMC3690640

[40] Vishwanathan R , Schalch W , Johnson EJ (2016) Macular pigment carotenoids in the retina and occipital cortex are related in humans. Nutr Neurosci 19, 95–101. 25752849 10.1179/1476830514Y.0000000141

[41] Nomura AM , Stemmermann GN , Lee J , Craft NE (1997) Serum micronutrients and prostate cancer in Japanese Americans in Hawaii. Cancer Epidemiol Biomarkers Prev 6, 487–491. 9232334

[42] Hastie T , Friedman J , Tibshirani R (2001) The Elements of Statistical Learning: Data Mining, Inference, and Prediction, Springer, New York.

[43] Christensen K , Gleason CE , Mares JA (2020) Dietary carotenoids and cognitive function among US adults, NHANES 2011-2014. Nutr Neurosci 23, 554–562. 30326796 10.1080/1028415X.2018.1533199 PMC6467741

[44] Mullan K , Cardwell CR , McGuinness B , Woodside JV , McKay GJ (2018) Plasma antioxidant status in patients with Alzheimer’s disease and cognitively intact elderly: A meta-analysis of case-control studies. J Alzheimers Dis 62, 305–317. 29439339 10.3233/JAD-170758

[45] Manochkumar J , Doss CGP , El-Seedi HR , Efferth T , Ramamoorthy S (2021) The neuroprotective potential of carotenoids *in vitro* and *in vivo*.. Phytomedicine 91, 153676. 34339943 10.1016/j.phymed.2021.153676

[46] Kabir MT , Rahman MH , Shah M , Jamiruddin MR , Basak D , Al-Harrasi A , Bhatia S , Ashraf GM , Najda A , El-Kott AF , Mohamed HRH , Al-Malky HS , Germoush MO , Altyar AE , Alwafai EB , Ghaboura N , Abdel-Daim MM (2022) Therapeutic promise of carotenoids as antioxidants and anti-inflammatory agents in neurodegenerative disorders. Biomed Pharmacother 146, 112610. 35062074 10.1016/j.biopha.2021.112610

[47] Hajizadeh-Sharafabad F , Zahabi ES , Malekahmadi M , Zarrin R , Alizadeh M AlizadehM(2021) Carotenoids supplementation and inflammation: A systematic review and meta-analysis of randomized clinical trials. Crit Rev Food Sci Nutr. doi: 10.1080/10408398.2021.1925870. 33998846

[48] Sachdeva AK , Chopra K (2015) Lycopene abrogates Abeta(1-42)-mediated neuroinflammatory cascade in an experimental model of Alzheimer’s disease. J Nutr Biochem 26, 736–744. 25869595 10.1016/j.jnutbio.2015.01.012

[49] Kim Y , Seo JH , Kim H (2011) beta-Carotene and lutein inhibit hydrogen peroxide-induced activation of NF-kappaB and IL-8 expression in gastric epithelial AGS cells. J Nutr Sci Vitaminol (Tokyo) 57, 216–223. 21908944 10.3177/jnsv.57.216

[50] Panasenko OM , Sharov VS , Briviba K , Sies H (2000) Interaction of peroxynitrite with carotenoids in human low density lipoproteins. Arch Biochem Biophys 373, 302–305. 10620353 10.1006/abbi.1999.1424

[51] Ghasemi M , Mayasi Y , Hannoun A , Eslami SM , Carandang R (2018) Nitric oxide and mitochondrial function in neurological diseases. Neuroscience 376, 48–71. 29462705 10.1016/j.neuroscience.2018.02.017

[52] Akbaraly NT , Faure H , Gourlet V , Favier A , Berr C (2007) Plasma carotenoid levels and cognitive performance in an elderly population: Results of the EVA Study. J Gerontol A Biol Sci Med Sci 62, 308–316. 17389729 10.1093/gerona/62.3.308

[53] Qu M , Shi H , Wang K , Wang X , Yu N , Guo B (2021) The associations of plasma/serum carotenoids with Alzheimer’s disease: A systematic review and meta-analysis. J Alzheimers Dis 82, 1055–1066. 34151808 10.3233/JAD-210384

[54] Zuniga KE , Bishop NJ , Turner AS (2021) Dietary lutein and zeaxanthin are associated with working memory in an older population. Public Health Nutr 24, 1708–1715. 32349832 10.1017/S1368980019005020 PMC10195438

[55] Wang W , Shinto L , Connor WE , Quinn JF (2008) Nutritional biomarkers in Alzheimer’s disease: The association between carotenoids, n-3 fatty acids, and dementia severity. J Alzheimers Dis 13, 31–38. 18334754 10.3233/jad-2008-13103

[56] Feeney J , O’Leary N , Moran R , O’Halloran AM , Nolan JM , Beatty S , Young IS , Kenny RA (2017) Plasma lutein and zeaxanthin are associated with better cognitive function across multiple domains in a large population-based sample of older adults: Findings from the Irish Longitudinal Study on Aging. J Gerontol A Biol Sci Med Sci 72, 1431–1436. 28329221 10.1093/gerona/glw330

[57] Vishwanathan R , Iannaccone A , Scott TM , Kritchevsky SB , Jennings BJ , Carboni G , Forma G , Satterfield S , Harris T , Johnson KC , Schalch W , Renzi LM , Rosano C , Johnson EJ (2014) Macular pigment optical density is related to cognitive function in older people. Age Ageing 43, 271–275. 24435852 10.1093/ageing/aft210 PMC3927776

[58] Feeney J , Finucane C , Savva GM , Cronin H , Beatty S , Nolan JM , Kenny RA (2013) Low macular pigment optical density is associated with lower cognitive performance in a large, population-based sample of older adults. Neurobiol Aging 34, 2449–2456. 23769396 10.1016/j.neurobiolaging.2013.05.007

[59] Mortamais M , Reynes C , Brickman AM , Provenzano FA , Muraskin J , Portet F , Berr C , Touchon J , Bonafe A , le Bars E , Maller JJ , Meslin C , Sabatier R , Ritchie K , Artero S (2013) Spatial distribution of cerebral white matter lesions predicts progression to mild cognitive impairment and dementia. PLoS One 8, e56972. 23457645 10.1371/journal.pone.0056972 PMC3572965

[60] Tokuchi R , Hishikawa N , Kurata T , Sato K , Kono S , Yamashita T , Deguchi K , Abe K (2014) Clinical and demographic predictors of mild cognitive impairment for converting to Alzheimer’s disease and reverting to normal cognition. J Neurol Sci 346, 288–292. 25248955 10.1016/j.jns.2014.09.012

[61] Strain JF , Barthelemy N , Horie K , Gordon BA , Kilgore C , Aschenbrenner A , Cruchaga C , Xiong C , Joseph-Mathurin N , Hassenstab J , Fagan AM , Li Y , Karch CM , Perrin RJ , Berman SB , Chhatwal JP , Graff-Radford NR , Mori H , Levin J , Noble JM , Allegri R , Schofield PR , Marcus DS , Holtzman DM , Morris JC , Benzinger TLS , McDade EM , Bateman RJ , Ances BM (2022) CSF Tau phosphorylation at Thr205 is associated with loss of white matter integrity in autosomal dominant Alzheimer disease. Neurobiol Dis 168, 105714. 35358703 10.1016/j.nbd.2022.105714 PMC9701560

[62] Power R , Nolan JM , Prado-Cabrero A , Roche W , Coen R , Power T , Mulcahy R (2022) Omega-3 fatty acid, carotenoid and vitamin E supplementation improves working memory in older adults: A randomised clinical trial. Clin Nutr 41, 405–414. 34999335 10.1016/j.clnu.2021.12.004

[63] Bone RA , Landrum JT , Cao Y , Howard AN , Alvarez-Calderon F (2007) Macular pigment response to a supplement containing meso-zeaxanthin, lutein and zeaxanthin. Nutr Metab (Lond) 4, 12. 17498306 10.1186/1743-7075-4-12 PMC1872023

[64] Nolan JM , Loskutova E , Howard A , Mulcahy R , Moran R , Stack J , Bolger M , Coen RF , Dennison J , Akuffo KO , Owens N , Power R , Thurnham D , Beatty S (2015) The impact of supplemental macular carotenoids in Alzheimer’s disease: A randomized clinical trial. J Alzheimers Dis 44, 1157–1169. 25408222 10.3233/JAD-142265

[65] Rasmussen HM , Muzhingi T , Eggert EMR , Johnson EJ (2012) Lutein, zeaxanthin, meso-zeaxanthin content in egg yolk and their absence in fish and seafood. Food Compost Anal 27, 139–144.

[66] Shyam R , Gorusupudi A , Nelson K , Horvath MP , Bernstein PS (2017) RPE65 has an additional function as the lutein to meso-zeaxanthin isomerase in the vertebrate eye. Proc Natl Acad Sci U S A 114, 10882–10887. 28874556 10.1073/pnas.1706332114 PMC5642693

[67] Thomson LR , Toyoda Y , Langner A , Delori FC , Garnett KM , Craft N , Nichols CR , Cheng KM , Dorey CK (2002) Elevated retinal zeaxanthin and prevention of light-induced photoreceptor cell death in quail. Invest Ophthalmol Vis Sci 43, 3538–3549. 12407166

[68] Bhosale P , Bernstein PS (2005) Synergistic effects of zeaxanthin and its binding protein in the prevention of lipid membrane oxidation. Biochim Biophys Acta 1740, 116–121. 15949677 10.1016/j.bbadis.2005.02.002

[69] Bhosale P , Larson AJ , Frederick JM , Southwick K , Thulin CD , Bernstein PS (2004) Identification and characterization of a Pi isoform of glutathione S-transferase (GSTP1) as a zeaxanthin-binding protein in the macula of the human eye. J Biol Chem 279, 49447–49454. 15355982 10.1074/jbc.M405334200

[70] O’Connell ML , Coppinger T , Lacey S , Arsenic T , McCarthy AL (2021) The nutritional status and dietary intake of free-living seniors: A cross-sectional study. Clin Nutr ESPEN 43, 478–486. 34024558 10.1016/j.clnesp.2021.02.020

[71] Morris MC , Evans DA , Tangney CC , Bienias JL , Wilson RS , Aggarwal NT , Scherr PA (2005) Relation of the tocopherol forms to incident Alzheimer disease and to cognitive change. Am J Clin Nutr 81, 508–514. 15699242 10.1093/ajcn.81.2.508

[72] de Leeuw FA , Schneider JA , Agrawal S , Leurgans SE , Morris MC (2020) Brain tocopherol levels are associated with lower activated microglia density in elderly human cortex. Alzheimers Dement (N Y) 6, 12021. 10.1002/trc2.12021 PMC7444784 32864412

[73] Browne D , McGuinness B , Woodside JV , McKay GJ (2019) Vitamin E and Alzheimer’s disease: What do we know so far? . Clin Interv Aging 14, 1303–1317. 31409980 10.2147/CIA.S186760 PMC6645610

[74] Huang HY , Appel LJ (2003) Supplementation of diets with alpha-tocopherol reduces serum concentrations of gamma- and delta-tocopherol in humans. J Nutr 133, 3137–3140. 14519797 10.1093/jn/133.10.3137

[75] Christen S , Woodall AA , Shigenaga MK , Southwell-Keely PT , Duncan MW , Ames BN (1997) gamma-Tocopherol traps mutagenic electrophiles such as NO(X) and complements alpha-tocopherol: Physiological implications. Proc Natl Acad Sci U S A 94, 3217–3222. 9096373 10.1073/pnas.94.7.3217 PMC20349

[76] Williamson KS , Gabbita SP , Mou S , West M , Pye QN , Markesbery WR , Cooney RV , Grammas P , Reimann-Philipp U , Floyd RA , Hensley K (2002) The nitration product 5-nitro-gamma-tocopherol is increased in the Alzheimer brain. Nitric Oxide 6, 221–227. 11890747 10.1006/niox.2001.0399

[77] Saint SE , Renzi-Hammond LM , Khan NA , Hillman CH , Frick JE , Hammond BR (2018) The macular carotenoids are associated with cognitive function in preadolescent children. Nutrients 10, 193. 29439387 10.3390/nu10020193 PMC5852769

[78] Mahmassani HA , Switkowski KM , Scott TM , Johnson EJ , Rifas-Shiman SL , Oken E , Jacques PF (2021) Maternal intake of lutein and zeaxanthin during pregnancy Is positively associated with offspring verbal intelligence and behavior regulation in mid-childhood in the Project Viva cohort. J Nutr 151, 615–627. 33484136 10.1093/jn/nxaa348 PMC7948203

[79] Grodstein F , Kang JH , Glynn RJ , Cook NR , Gaziano JM (2007) A randomized trial of beta carotene supplementation and cognitive function in men: The Physicians’ Health Study II. Arch Intern Med 167, 2184–2190. 17998490 10.1001/archinte.167.20.2184

[80] Johnson EJ , McDonald K , Caldarella SM , Chung HY , Troen AM , Snodderly DM (2008) Cognitive findings of an exploratory trial of docosahexaenoic acid and lutein supplementation in older women. Nutr Neurosci 11, 75–83. 18510807 10.1179/147683008X301450

[81] Hammond BR , Jr. , Miller LS , Bello MO , Lindbergh CA , Mewborn C , Renzi-Hammond LM (2017) Effects of lutein/zeaxanthin supplementation on the cognitive function of community dwelling older adults: A randomized, double-masked, placebo-controlled trial. Front Aging Neurosci 9, 254. 28824416 10.3389/fnagi.2017.00254 PMC5540884

[82] Renzi-Hammond LM , Bovier ER , Fletcher LM , Miller LS , Mewborn CM , Lindbergh CA , Baxter JH , Hammond BR (2017) Effects of lutein/zeaxanthin intervention on cognitive function: A randomized, double-masked, placebo-controlled trial of younger healthy adults. Nutrients 9, 9111246. 10.3390/nu9111246 PMC5707718 29135938

[83] Power R , Coen RF , Beatty S , Mulcahy R , Moran R , Stack J , Howard AN , Nolan JM (2018) Supplemental retinal carotenoids enhance memory in healthy individuals with low levels of macular pigment in a randomized, double-blind, placebo-controlled clinical trial. J Alzheimers Dis 61, 947–961. 29332050 10.3233/JAD-170713

[84] Stringham NT , Holmes PV , Stringham JM (2019) Effects of macular xanthophyll supplementation on brain-derived neurotrophic factor, pro-inflammatory cytokines, and cognitive performance. Physiol Behav 211, 112650. 31425700 10.1016/j.physbeh.2019.112650

[85] Bovier ER , Renzi LM , Hammond BR (2014) A double-blind, placebo-controlled study on the effects of lutein and zeaxanthin on neural processing speed and efficiency. PLoS One 9, 108178. 10.1371/journal.pone.0108178 PMC4176961 25251377

[86] Ceravolo SA , Hammond BR , Oliver W , Clementz B , Miller LS , Renzi-Hammond LM (2019) Dietary carotenoids lutein and zeaxanthin change brain activation in older adult participants: A randomized, double-masked, placebo-controlled trial. Mol Nutr Food Res 63, e1801051. 30950580 10.1002/mnfr.201801051

[87] Lindbergh CA , Renzi-Hammond LM , Hammond BR , Terry DP , Mewborn CM , Puente AN , Miller LS (2018) Lutein and zeaxanthin influence brain function in older adults: A randomized controlled trial. J Int Neuropsychol Soc 24, 77–90. 28695791 10.1017/S1355617717000534

[88] Mullan K , Williams MA , Cardwell CR , McGuinness B , Passmore P , Silvestri G , Woodside JV , McKay GJ (2017) Serum concentrations of vitamin E and carotenoids are altered in Alzheimer’s disease: A case-control study. Alzheimers Dement (N Y) 3, 432–439. 29067349 10.1016/j.trci.2017.06.006 PMC5651431

[89] Tanprasertsuk J , Mohn ES , Matthan NR , Lichtenstein AH , Barger K , Vishwanathan R , Johnson MA , Poon LW , Johnson EJ (2019) Serum carotenoids, tocopherols, total n-3 polyunsaturated fatty acids, and n-6/n-3 polyunsaturated fatty acid ratio reflect brain concentrations in a cohort of centenarians. J Gerontol A Biol Sci Med Sci 74, 306–314. 29893813 10.1093/gerona/gly125

[90] Schwarzinger M , Pollock BG , Hasan OSM , Dufouil C , Rehm J , QalyDays Study G (2018) Contribution of alcohol use disorders to the burden of dementia in France 2008-13: A nationwide retrospective cohort study. Lancet Public Health 3, e124–e132. 29475810 10.1016/S2468-2667(18)30022-7

[91] Stahl W , Junghans A , de Boer B , Driomina ES , Briviba K , Sies H (1998) Carotenoid mixtures protect multilamellar liposomes against oxidative damage: Synergistic effects of lycopene and lutein. FEBS Lett 427, 305–308. 9607334 10.1016/s0014-5793(98)00434-7

[92] Mey J (2017) RAR/RXR-mediated signaling. In Gene Regulation, Epigenetics and Hormone Signaling, Mandal PSS, ed. Wiley-VCH Verlag GmbH &Co., KGaA, pp. 457-510.

[93] Clark JN , Whiting A , McCaffery P (2020) Retinoic acid receptor-targeted drugs in neurodegenerative disease. Expert Opin Drug Metab Toxicol 16, 1097–1108. 32799572 10.1080/17425255.2020.1811232

[94] Zou X , Gao J , Zheng Y , Wang X , Chen C , Cao K , Xu J , Li Y , Lu W , Liu J , Feng Z (2014) Zeaxanthin induces Nrf2-mediated phase II enzymes in protection of cell death. Cell Death Dis 5, 190. 10.1038/cddis.2014.190 PMC4047913 24810054

[95] Chowdhury I , Mo Y , Gao L , Kazi A , Fisher AB , Feinstein SI (2009) Oxidant stress stimulates expression of the human peroxiredoxin 6 gene by a transcriptional mechanism involving an antioxidant response element. Free Radic Biol Med 46, 146–153. 18973804 10.1016/reeradbiomed.2008.09.027 PMC2646855

[96] Fisher AB (2017) Peroxiredoxin 6 in the repair of peroxidized cell membranes and cell signaling. Arch Biochem Biophys 617, 68–83. 27932289 10.1016/j.abb.2016.12.003 PMC5810417

[97] Manevich Y , Hutchens S , Tew KD , Townsend DM (2013) Allelic variants of glutathione S-transferase P1-1 differentially mediate the peroxidase function of peroxiredoxin VI and alter membrane lipid peroxidation. Free Radic Biol Med 54, 62–70. 23142420 10.1016/j.freeradbiomed.2012.10.556 PMC3539142

[98] Bradley-Whitman MA , Lovell MA (2015) Biomarkers of lipid peroxidation in Alzheimer disease (AD): An update. Arch Toxicol 89, 1035–1044. 25895140 10.1007/s00204-015-1517-6 PMC4466146

[99] Pinhel MA , Nakazone MA , Cacao JC , Piteri RC , Dantas RT , Godoy MF , Godoy MR , Tognola WA , Conforti-Froes ND , Souza D (2008) Glutathione S-transferase variants increase susceptibility for late-onset Alzheimer’s disease: Association study and relationship with apolipoprotein E epsilon4 allele. Clin Chem Lab Med 46, 439–445. 18298341 10.1515/CCLM.2008.102

[100] Lim BP , Nagao A , Terao J , Tanaka K , Suzuki T , Takama K (1992) Antioxidant activity of xanthophylls on peroxyl radical-mediated phospholipid peroxidation. Biochim Biophys Acta 1126, 178–184. 1627620 10.1016/0005-2760(92)90288-7

[101] Scheidegger R , Pande AK , Bounds PL , Koppenol WH (1998) The reaction of peroxynitrite with zeaxanthin. Nitric Oxide 2, 8–16. 9706738 10.1006/niox.1997.0156

[102] Yoshioka R , Hayakawa T , Ishizuka K , Kulkarni A , Terada Y , Maoka T , Etoh H (2006) Nitration reactions of astaxanthin and b-carotene by peroxynitrite. Tetrahedron Lett 47, 3637–3640.

[103] Suzuki R , Kulkarni A , Yomoda Y , Kawagishi H , Terada Y , Maoka T , Etoh H (2007) Reaction of retinol with peroxynitrite. Biosci Biotechnol Biochem 71, 2596–2599. 17928683 10.1271/bbb.70349

[104] Maoka T , Tokuda H , Suzuki N , Kato H , Etoh H (2012) Anti-oxidative, anti-tumor-promoting, and anti-carcinogensis activities of nitroastaxanthin and nitrolutein, the reaction products of astaxanthin and lutein with peroxynitrite. Mar Drugs 10, 1391–1399. 22822380 10.3390/md10061391 PMC3397447

[105] Bhosale P , Zhao DY , Serban B , Bernstein PS (2007) Identification of 3-methoxyzeaxanthin as a novel age-related carotenoid metabolite in the human macula. Invest Ophthalmol Vis Sci 48, 1435–1440. 17389468 10.1167/iovs.06-1046

[106] Everett SA , Dennis MF , Patel KB , Maddix S , Kundu SC , Willson RL (1996) Scavenging of nitrogen dioxide, thiyl, and sulfonyl free radicals by the nutritional antioxidant beta-carotene. J Biol Chem 271, 3988–3994. 8626730 10.1074/jbc.271.8.3988

[107] Mortensen A (2002) Scavenging of benzylperoxyl radicals by carotenoids. Free Radic Res 36, 211–216. 11999390 10.1080/10715760290006501

[108] Jimenez-Jimenez FJ , Molina JA , de Bustos F , Orti-Pareja M , Benito-Leon J , Tallon-Barranco A , Gasalla T , Porta J , Arenas J (1999) Serum levels of beta-carotene, alpha-carotene and vitamin A in patients with Alzheimer’s disease. Eur J Neurol 6, 495–497. 10362906 10.1046/j.1468-1331.1999.640495.x

[109] Rinaldi P , Polidori MC , Metastasio A , Mariani E , Mattioli P , Cherubini A , Catani M , Cecchetti R , Senin U , Mecocci P (2003) Plasma antioxidants are similarly depleted in mild cognitive impairment and in Alzheimer’s disease. Neurobiol Aging 24, 915–919. 12928050 10.1016/s0197-4580(03)00031-9

[110] Zaman Z , Roche S , Fielden P , Frost PG , Niriella DC , Cayley AC (1992) Plasma concentrations of vitamins A and E and carotenoids in Alzheimer’s disease. Age Ageing 21, 91–94. 1575097 10.1093/ageing/21.2.91

